# Animal welfare and sustainability in pork production: a pilot study of farmer and veterinarian perspectives in North America

**DOI:** 10.1186/s40813-026-00521-5

**Published:** 2026-05-30

**Authors:** Martyna E. Lagoda, Kyle A. T. Moak, Kayla Arisman, Melanie Kaczur, Thaisa L. Sandri, Niels Wuyts, Leanne Van De Weyer, Yolande M. Seddon

**Affiliations:** 1 Western College of Veterinary Medicine, University of Saskatchewan, Saskatoon, Canada; 2 Canadian Hub for Applied and Social Research, University of Saskatchewan, Saskatoon, Canada; 3 Zoetis, Global Medical Affairs, 10 Sylvan Way, Parsippany, NJ 07054 USA; 4 Zoetis Canada, 16, 740 Trans-Canada, Kirkland, QC Canada

**Keywords:** Swine, Well-being, Sustainability, Stakeholder perceptions, Management practices

## Abstract

**Background:**

The swine industry is under increasing pressure to adopt meaningful animal welfare improvement strategies, yet implementation remains challenging due to competing operational demands and the economic costs associated with many welfare interventions in a pork market that is often volatile. Although good animal welfare is known to enhance productivity, profitability, and long-term sustainability, clearer stakeholder understanding of these relationships is needed to support industry decision making for broader adoption of welfare strategies. This pilot study examined how pork industry stakeholders perceive animal welfare and its role in business decisions related to sustainable production by assessing: [1] how producers and swine veterinarians define animal welfare and the metrics they use to monitor it on-farm; [2] stakeholder understanding of how management procedures influence welfare, whether decisions are driven by welfare or productivity concerns, and whether barriers to implementation exist; and [3] stakeholder perceptions of the relationships between welfare and broader sustainability metrics.

**Results:**

Concept elicitation interviews with open-ended questions were conducted to capture firsthand stakeholder experiences with on-farm welfare management. Ten participants were interviewed; four experienced swine managers and six veterinarians, including seven from Canada and three from the United States. Interviews elicited detailed descriptions of participants’ definitions of animal welfare, the management practices they implemented and the motivations behind management decisions, welfare and productivity indicators used, perceived impacts of management practices on staff well-being, and views on the interconnections between welfare, production performance, and overall business sustainability. Producers generally did not provide a clear definition of animal welfare, but referenced welfare indicators they use to assess welfare; these being primarily visible, physical measures related to economically relevant performance metrics. Respondents acknowledged the value of good welfare for profitability and staff well-being, however, welfare considerations typically remained secondary to economic priorities, and practices involving upfront costs were rarely adopted unless mandated.

**Conclusions:**

This study highlights that although animal welfare is widely recognized as important to sustainable pork production, its role is not yet fully understood or embedded within the workplace culture or business models of pork production systems.

**Supplementary Information:**

The online version contains supplementary material available at 10.1186/s40813-026-00521-5.

## Background

In pork production, business sustainability refers to the sector’s ability to remain economically viable over the long term by maintaining efficiency, whilst producing and rearing animals with ethical responsibility that aligns with societal expectations, and minimizing the environmental footprint of production [1]. However, the swine industry is subject to a range of economic, societal, and ethical pressures that challenge its long-term sustainability [2]. Global trade exerts downward pressure on pork prices, favouring the lowest cost of production and perpetuating inadequate compensation for producers in higher-cost regions. Rising input costs, particularly for feed, coupled with persistent demand for low-cost animal protein and environmental sustainability targets contribute to persistently narrow profit margins [2]. The physically demanding nature of pork production occupations combined with unappealing working environments that are often characterized by poor air quality, remote locations, social isolation, and low pay contribute to labour challenges which further constrain the capacity for systemic improvements [3]. These limitations are of particular concern given the growing societal demand for enhanced animal welfare practices and a clearer understanding of how food animals are raised and managed in modern production systems [2, 4]. The swine industry needs to incorporate improvements to animal welfare into their operations whilst balancing other areas of their business model required for sustainability.

The Five Freedoms (5Fs) is a globally recognized framework that continues to serve as a benchmark standard for companies seeking to meet animal welfare requirements [5]. Capturing (1) freedom from hunger and thirst, (2) freedom from discomfort, (3) freedom from pain, injury, and disease, (4) freedom from fear and distress, and (5) freedom to express natural behaviour [6], it provides a comprehensive guide to the fundamental conditions required for good animal welfare. Understanding how stakeholders responsible for animal care, such as pork producers and veterinarians, interpret animal welfare and apply management practices in the context of the 5Fs can help identify the barriers and opportunities for improving welfare in a way that supports the long-term viability of the swine industry.

A substantial body of scientific evidence supports the link between animal welfare, productivity, and sustainable practices in pork production [7–10]. For example, physically and psychologically healthy animals are more resilient to disease [11, 12], which can reduce veterinary treatment costs, antimicrobial usage, morbidity and mortality rates [7, 13–15]. Improvements in welfare can also enhance performance indicators such as improved average daily gain and feed conversion ratio, contributing to a more efficient production with improved economic and environmental sustainability [16].

Despite these benefits, the long-term economic advantages of good welfare are often not immediately apparent. Additionally, animal welfare improvement strategies can be costly and require human behaviour change at the farm level [17, 18]. For example, increasing the space allowance requires either infrastructure changes to barn designs, which is a significant cost, or a reduction in herd size, affecting farm production throughput and the subsequent economic capabilities. Providing pigs with environmental enrichment increases overhead costs through the enrichment materials and labour to provide and replace it [17]. With narrow profit margins, these costs can discourage producers from making investments to improve animal welfare [17]. A scarcity of economic analyses of the cost-benefit of welfare strategies in scientific literature exacerbates this further creating a barrier to evidence-based decision-making for producers and contributes to resistance towards the adoption of animal welfare-enhancing strategies [4, 17].

A poor understanding of animal welfare, and inconsistencies in the interpretation by different stakeholders can contribute to resistance and a failure to consider the role of welfare in sustainable production practices [19]. Pork producers often equate animal welfare with basic care - feeding, health checks, and veterinary treatment, while overlooking pigs’ psychological and behavioural needs [2]. While veterinarians receive more formal welfare training than producers, their focus still tends to centre on physical health and veterinary treatment needs [2]. A lack of shared understanding between stakeholders regarding what constitutes good welfare can undermine the perceived necessity or value of welfare-enhancing practices in sustainable production practices, impeding the integration of animal welfare into company culture [20]. Some producers also view animal welfare through a regulatory lens, seeing it as a series of requirements that introduce additional costs [21]. When welfare initiatives are perceived as imposed rather than collaboratively developed, this can foster resistance and limit engagement with welfare strategies.

To advance sustainable pork production practices, it is essential to understand how pork industry stakeholders perceive animal welfare and its role in business decisions around sustainable pork production. Through interviews with pork sector stakeholders, the objectives of this pilot study were (1) to assess how pork producers and swine veterinarians define animal welfare and the metrics they use to monitor welfare on-farm; (2) to assess stakeholder understanding on how their farm management procedures influence animal welfare, and identify whether animal management decisions were made based on welfare or productivity, or whether barriers to implementation exist; and (3) to assess stakeholder understanding of the relationships between animal welfare and broader pork production sustainability metrics. This knowledge has the potential to be used to improve the implementation of the 5Fs concept within the business sustainability model for stakeholders.

## Methods

This study was approved by the University of Saskatchewan behavioural research ethics board for data collected from living human participants, ID: #4154.

### Recruitment of participants

Recruitment strategies were consistent for participants in Canada and the United States (U.S.) and involved communication of an invitation to participate in the research via direct e-mail contact with professional connections, communication through pork councils, industry events (communicated to meeting organisers and/or Y. Seddon in-person communication to individuals at industry events), social media channels, and chain referrals. Respondents were all recruited through direct contact (most commonly e-mail, followed up with a phone call). In at least two companies that participated, they selected appropriate staff to participate based on their experience relevant to the goals of the interview (i.e.: were responsible for decision making on animal care). Eligible participants included pig production managers or swine veterinarians responsible for providing advice and making decisions on animal care and/or pork business decisions. The recruitment process concluded after 10 individuals (seven from Canada, and three from the U.S.) were enrolled, and, due to recruitment challenges, was concluded within one year (2024). Of the individuals that were recruited 6 knew of Dr. Seddon professionally through her research in Canada, 3 were recruited through selection by their company, of which each company knew Dr. Seddon professionally, and 1 individual participated following an in-person invitation to participate from Dr. Seddon at a pork industry event. This individual did not have a professional connection to Dr. Seddon.

### Interview design and process

A concept elicitation interview with open-ended questions was designed to capture the first-hand experience of participants in applying on-farm animal welfare management strategies. In doing so, it explored the links between animal welfare, productivity, and broader sustainability goals, defined as the ability of pork production systems to remain economically viable while balancing efficiency, resilience, and responsibility toward animals, staff, the environment, and society. Hence, a semi-structured interview guide was developed for systematically obtaining direct recalled observations from the participants [22]. This approach was chosen to encourage participants to draw on their practical experience and support their thinking process. The interview (see Additional file 1 for the interview script) consisted of six sections including questions regarding the participants’ background and demographics, work experience, animal welfare and its on-farm indicators, management procedures and their relationship to animal welfare and production performance, influence of animal management on staff well-being, and productivity measures and their relationship to animal welfare and sustainability. The interview aimed to elicit in-depth descriptions of how participants understood and defined animal welfare, which management procedures they implemented and why, what indicators they used to monitor animal welfare and productivity, how management procedures affected staff well-being, and how they perceived the interrelationships between animal welfare, production performance, and the overall business sustainability goals of their operations. The interview questions were piloted on three members of the University of Saskatchewan’s Prairie Swine Center (research and barn staff), following which, the phrasing of a few select questions was changed to improve clarity for interviewees on what was being asked to support focus on the topic of animal welfare in the production environment, but the main structure and topic areas remained the same. Except for the sections covering background and work experience, open-ended questions were used along with follow-up prompts to facilitate free discussion as needed.

Recruited participants signed a written consent form before the start of the interview confirming agreement to have their audio, and where applicable, video recorded. Participants were informed that their participation was voluntary, that they could refuse to answer any question, and that they had the right to withdraw from the study at any time and their data would be erased. Interviews were conducted by two interviewers using either Zoom (Zoom Video Communications, Inc., CA, U.S.A.), recorded through Zoom’s in-house recording software, and with a back-up recorded using a voice recorder (Evistr Digital Voice Recorder, DongGuan Wista Electronics Co., LTD, China), or over the phone, recorded with a voice recorder (Evistr Digital Voice Recorder, as above). All interviews took place between January and October 2024 and ranged from 46 to 128 min.

### Data storage and analysis

Interviews were transcribed verbatim by the same two individuals who conducted the interviews. Participants were provided with their interview transcript and were given one week to revise their answers, or add further comments or edits. Hard-copy data (i.e.: physically signed consent forms) were stored in a locked filing cabinet at the University of Saskatchewan. Electronic data (i.e.: recordings, identifying information, and consent forms) were stored on a secure University of Saskatchewan server with access restricted to designated research personnel. Audio and video recordings were erased once the information was transcribed. Both physical and electronic data were retained for a maximum of five years following collection, unless otherwise requested by the interviewee, and after this period, all material would be destroyed beyond recovery. Participant personal data were stored separately from transcript data, and all transcripts were anonymized to ensure participant confidentiality and anonymity.

Anonymized qualitative data from the reviewed transcripts were imported and analyzed in NVivo Version 12 for Windows and Mac (QSR International Pty Ltd., Melbourne, Australia). Inductive thematic coding was performed by an analyst from the University of Saskatchewan’s Canadian Hub for Applied and Social Research (CHASR) to identify thematic elements in the responses. Briefly, inductive thematic coding is the process of developing codes and categorizing them into themes that are identified from the raw data, without having a preexisting coding theme by iteratively reevaluating the data and grouping common responses into themes [23]. The benefit of this approach is that identified themes have a strong relationship to the narratives of the interviewed participants, are grounded in participant understanding of concepts, and are data-driven [23]. The analyst engaged in repeated reading of the data to ensure immersion and to begin generating initial impressions. During this phase, preliminary notes were recorded to capture early ideas and potential areas of interest. Broad-level codes were generated inductively as they appeared in the data, and related codes were then grouped iteratively into potential themes. All themes were reviewed and refined across the entire dataset to ensure they were consistent with the theme labels. Larger thematic areas were broken down into sub-themes if they were multifaceted and warranted further differentiation. One of the interviewers familiar with pork production then screened the thematic analysis to ensure that theme labels were appropriate, and that the analysis comprehensively captured meaningful patterns in the data. The interview data were not stratified by profession or nationality due to the limited number of participants in each group. Representative quotes were selected from participant interviews to illustrate and support key themes in relation to the study objectives. Excerpts from veterinarians (**V**) are bolded, and those from producers (*P*) are in simple italics. Participant demographic data were summarised descriptively.

## Results

### Sample size and respondent demographics

Concept saturation, defined as the point at which no new relevant information emerges [23], was reached for certain discussion areas such as productivity indicators, despite the low number of participants (*n* = 10) in this study. Although Guest et al. [24] indicate that saturation is often achieved within the first 12 interviews, the diversity of respondents interviewed (including veterinarians and producers with varying levels of experience and from different nationalities) likely provided sufficient variation in perspectives to achieve concept saturation within 10 interviews. Nevertheless, given the small sample size, we cannot confidently claim that the results are fully representative.

In total, four experienced swine managers (producers) and six veterinarians were interviewed, with seven respondents from Canada and three from the U.S., (Table 1).

**Table 1:** Demographics of respondents who participated in an interview on animal welfare and business sustainability goals

Demographics	Producers	Veterinarians
Professional education		
Bachelor’s degree	3	-
College diploma	1	-
Doctor of Veterinary Medicine	-	5
Veterinary diploma	-	1
Role title		
Consulting swine veterinarian	-	1
Director of health (veterinarian)	-	2
Director of technical services	1	-
Production director or production manager	3	-
Swine veterinarian	-	3
Years of experience		
Less than 5	-	-
6 to 20	4	6
20+	-	-
Management role		
No management involvement	-	1
Some management involvement	-	3
Primarily management involvement	4	2
Prior experience working with pigs		
No	-	-
Yes	4	6
Stage of specialization		
All stages	3	6
Farrowing	1	-
Finishing	-	-
Growing	-	-
Nursery	-	-
Responsible for meeting animal welfare and sustainability goals		
Yes	4	5
Undecided^1^	-	1
Experience with other animals		
No	2	1
Yes	2	3
Missing^2^	-	2

^1^ Refers to an inconclusive response

^2^ Refers to participants who were not asked this question

### Stakeholder understanding of animal welfare

The interview began with respondents being asked about their understanding of animal welfare, and specifically, what animal welfare meant to them. Neither producers nor veterinarians explained animal welfare using a standard, recognised definition. Instead, respondents described components of what good welfare should entail, or how it was implemented within their systems. One veterinarian provided their own definition of animal welfare, which made reference to shelter, feed and water, veterinary treatments, no fear, and humane euthanasia, but omitted the importance of freedom to express natural behaviour. Many aligned good animal welfare to treating pigs with respect and empathy (“*It’s just about treating the animals with respect. Giving them everything that they require*”), but acknowledged that this is limited by the constraints of the system (“*Giving the pigs the best life possible. Within financial constraints and business constraints*”).

In describing what animal welfare means to them, the 5Fs framework was mentioned either explicitly (*P* = 1, V = 3), or implicitly (*P* = 3, V = 2) by respondents (“*Making sure that all the five freedoms are met in all aspects of our animal care. So*,* they basically become the underpinning or the foundation for the decisions that we make*”; “***Meeting basic needs, (…) making sure the animals have feed and water, and environment is okay for them. Not being abused is a big one”***). Freedom from thirst and hunger was mentioned most often (P = 3, V = 5). Respondents understood the direct link between physiological needs of an animal and productivity (“*Obviously food and water… Providing everything that they need from a nutritional and maintenance standpoint to make sure they produce for us as well as they can*”). Freedom from discomfort was the second most mentioned aspect of the 5Fs (*P* = 2, V = 4), and was mentioned in the context of the physiological needs of an animal, i.e.: the need for shelter and comfortable resting areas. This highlights that the stakeholders intuitively associate good animal welfare with satisfying the physiological needs of animals. In their understanding of animal welfare, freedom from disease, pain and injury was mentioned the least among participants (*P* = 1, V = 2). This was the case despite health concerns being mentioned as a major challenge to animal welfare and productivity, and visible signs of disease, pain, and injury being one of the main animal-based indicators used by respondents to monitor welfare. Freedom from fear and distress was mentioned as important to consider for animal welfare by half of the participants (*P* = 2, V = 3), and the freedom to express natural behaviour was mentioned by only one producer, but three veterinarians.

Respondents reported that they most commonly monitored welfare using animal-based indicators, particularly visible, physical signs of compromised welfare (i.e.: poor body condition, hernias), pain behaviour, disease and lesions on the body indicative of injury (shoulder sores), damaging (tail biting) and aggressive (skin lesions) behaviours. The number of treatments and the number of positive outcomes resulting from treatments, as well as the number of pigs euthanized versus found dead, and mortality rates were measures also used to monitor welfare. Although less common, respondents also reported using behaviour to monitor welfare, including the responses of pigs towards humans (fearful or positive/calm), levels of arousal in the pen, i.e.: resting after feeding as a sign of comfort and good social dynamics, and presence of stereotypical behaviours.

### Perception of the relationship between management procedures and animal welfare

Respondents were then asked to speak about the management procedures that are in place in the systems they work within (Table 2), the rationale behind each procedure, and how they considered each procedure impacts the welfare of pigs.

**Table 2:** Frequency/percentage of respondents (*n* = 10) reporting the use [always/yes); never/no; or occasionally] of each management procedure

Existing management procedures	Yes	No	Occasionally
Immunocastration	8 (80%)	2 (20%)	4 (40%)^1^
Surgical castration	6 (60%)	4 (40%)	4 (40%)^1^
Tail docking	10 (100%)	0 (0%)	0 (0%)
Use of pain management at processing	8 (80%)	2 (20%)	0 (0%)
Mixing of both sexes in groups	3 (30%)	3 (30%)	4 (40%)^1^
Maintaining same social group throughout production	9 (90%)	0 (0%)	1 (10%)^1^
Provision of environmental enrichment	8 (80%)	1 (10%)	1 (10%)^1^

^1^ Refers to producers who use immunocastration only for cryptorchid piglets; for veterinarian respondents, it denotes those working with clients who practice both procedures, and therefore also appear in the “yes” category giving rise to an inflated total (> 100%)

All respondents reported following quantitative industry recommendations for space allocation, while 90% stated they adhered to recommendations for resource allocation (feed and water); the remaining 10% did not comment.

Among routine management practices, castration and tail docking are recognised as painful procedures [25]. Surgical castration is performed on male piglets in the first week of life (3 to 7 days of age) to remove the testes and prevent the development of boar taint in the meat. Immunocastration is used as an alternative to surgical castration. It involves vaccinating pigs against gonadotropin-releasing hormone which controls testicular development and function, thus preventing boar taint development while avoiding the pain and stress associated with surgery. Tail docking is performed on piglets to reduce the risk of tail biting, a damaging behaviour commonly observed in intensive pig production systems which results in injuries, infections, and is reflective of compromised welfare.

Surgical castration was a standard practice reported by two (20%) respondents (*P* = 1, V = 1), while four (40%) respondents (*P* = 2, V = 2) reported immunocastration, and four (40%) (*P* = 1, V = 3) occasionally used immunocastration. In these latter cases, three veterinarians represented clients that practiced surgical and some immunocastration. One producer primarily performed surgical castration, with immunocastration used only on pigs with undescended testes (cryptorchid piglets). All respondents practiced tail docking.

### Reasoning for the management decisions and consideration for animal welfare impacts

The decision to adopt immunocastration was rarely framed as being primarily motivated by benefits to animal welfare with only two (20%) respondents referring to animal welfare as an indirect consideration. One veterinarian stated that management choices generally balance economic returns with animal welfare (“***Sometimes we have to do it because it’s the right thing to do for the pigs. But it might not always have a direct correlation to revenue***”), while one producer stated that immunocastration “*[It’s] just an injection. It’s just like taking a Covid or flu vaccine for us. It’s no different [to a vaccination]. So immunocastration it doesn’t need to have any pain control*”, suggesting an acknowledgment that surgical castration is painful for pigs and that immunocastration eliminates this pain, thereby removing the need for pain control. However, explicit concern for the pig’s welfare was not expressed in this context. Respondents who did not or only occasionally used immunocastration stated they were limited in their ability to use it by the market/abattoir requirements (*P* = 1, V = 1). Two veterinarians (representing clients who never or occasionally used immunocastration) stated that surgical castration was performed to prevent boar taint and improve the taste of meat. One producer noted they had no control over farm practices but would personally choose immunocastration for its labour-saving benefits, underscoring that productivity and efficiency, rather than welfare, are often the main drivers of decisions.

In the case of tail docking, eight (80%; *P* = 4, V = 4) respondents explained this procedure was practiced to prevent tail biting (“*For tail docking, it is strictly for the nursery-finisher to prevent tail biting and issues there*”). At least four (*P* = 2, V = 2) respondents justified tail docking by weighing its short-term negative welfare impacts against potential long-term benefits to welfare resulting from reduced tail biting (“***You’re balancing one welfare concern with another. It’s a stressor, but it’s the lesser of two evils”; “If you don’t dock, then later in life, they’re highly likely to have a pen mate chew it off***”). This suggests that with regards to tail-docking, welfare implications were considered to some extent in the decision-making process for performing this management procedure.

Although welfare was not stated as the primary motivation by 80% of respondents, 30% of respondents (*P* = 2, V = 1) who always or occasionally used immunocastration acknowledged it as better for animal welfare, being less painful and carrying lower infection risk (“***If we have these other options, like [immunocastration], vaccination is better, no question, from a welfare standpoint***”; “*Eliminating physical castration has been a key improvement in animal welfare. Any time you’re not cutting two holes in the back of an animal it’s a good thing*”). Two producers added that use of immunocastration seemed to be associated with calmer pigs, less tail biting, and larger barrows. However, one veterinarian (representing clients who occasionally use immunocastration) raised concerns about increased mounting behaviour and possible leg fractures. One producer and one veterinarian who performed surgical castration stated it did not appear to negatively affect piglets and considered both methods equally beneficial for welfare. Similarly, 30% of the respondents (*P* = 2, V = 1) regarded tail docking as having minimal negative impact on pigs (“***Pigs typically return to nursing within seconds to minutes after having a procedure done***”). One producer also suggested that tail docking benefits piglets by providing staff an opportunity to inspect and handle each animal.

#### Pain management during painful procedures

Provision of pain control (anesthetic and/or analgesia) at the time of painful procedures is a crucial step in supporting better welfare. It is a requirement of the Canadian Pig Code of Practice for the Care and Handling of Pigs [26]. However, it must be noted that providing analgesia at the time of the procedure does not address pain at the point of castration, nor the full extent of post-procedural pain during the period of acute inflammation that lasts 3–5 days following tissue injury. Eight (80%) respondents indicated that they use pain management at the time of piglet processing. Notably, respondents who stated that pain management is not used on their farms for either tail docking or surgical castration were from the U.S., where no pain control is required in industry guidelines or approved for use in pigs [27]. For those who provide pain management, six (60%) respondents indicated their decision was based on following industry guidelines; the Canadian Code of Practice for the Care and Handling of Pigs, or based on the contract signed with the abattoir. Of the respondents who indicated that they do not provide analgesia, one stated that they do not follow any guidelines, and have not been given any direction on using pain control for processing. This indicates that, in the absence of mandated requirements, there is limited motivation or initiative within companies to implement pain mitigation strategies for routine painful procedures, suggesting that the economic considerations such as reduced labour needs and lower medication costs are prioritized over the welfare of pigs.

#### Management of tail biting

Nine (90%) respondents had experience with tail biting in their herd, and one (10%) respondent from the U.S. indicated they had not seen tail biting in their barn over the last five years at the time of the interview. When asked to reflect on potential changes in pig behaviour prior to tail biting outbreaks, none of the respondents indicated that they observed changes to behaviour, however, one veterinarian indicated they may occasionally see flank biting prior to tail biting. Furthermore, only one (10%) producer made the connection between tail biting and the natural behaviour needs of pigs (“*There is that instinct to root and chewing behaviour with pigs. It’s what they were bred to do out in the woods finding their sustenance*”). Four (40%) respondents demonstrated an understanding of the multifactorial nature of tail biting (“***Tail biting is complex in the sense that there is multiple different components that are contributing***”), while the remaining respondents did not make this connection. Several procedures were mentioned when asked about the management of a tail biting outbreak that has already started. These included the removal of the instigating pig or victims of biting (70%), an increase in ventilation (60%), diet adjustments (50%), increased access to feed and water (50%), a review of the environment (40%), addition of enrichment (30%), the use of commercially available anti-chewing sprays (30%), testing of feed (30%), reduction of temperature (20%), decreasing the stocking density (20%), consulting with veterinarians (10%), cleaning of pens (10%), or remixing pigs (10%). Given the large number of management strategies discussed, respondents understood the need for systematic approaches, including checklists and evidence-based management techniques to prevent and address tail biting effectively. In the discussion of the links between tail biting and welfare, only half (50%) of the respondents reflected on tail biting being an indicator of broader problems related to animal welfare, citing discomfort, stress and disease as the main factors (“*I suspect that there is something maybe around welfare, that’s the broadest sense, in terms of the one doing the biting as well. It’s saying it’s not happy with something. There’s some sort of stressor in there, that’s my suspicion*”).

#### Management of compromised animals

The main procedures used in relation to the management of sick animals included the use of hospital pens (100% of respondents), antibiotics or medication (40%), euthanasia (30%), provision of supplemental feed and warmer environments (20%), regular checks on animals (20%), and identification of sick animals for treatment by tagging (20%). Two veterinarians (20%) also cited the use of treatment records or comfort mats. Although participants did not explicitly link these practices to animal welfare, their use suggests an effort to prevent sickness, disease, and pain. Respondents were not directly asked to comment on what was the motivation for hospital pen use in the treatment of sick animals, and consequently responses lacked context on whether decisions were driven by welfare concerns, productivity gains, or to mitigate loss.

#### Maintaining same social groups throughout production

This management procedure is performed to minimize stress, aggression and injury associated with mixing of unfamiliar pigs. Most respondents (90%) indicated that they aimed to maintain pigs in the same social groups throughout production. However, it was recognized that this is not always possible, especially when transferring pigs from the nursery to the grower stage. The main reasons for keeping pigs in the same groups appeared to be the need to implement specific production practices, whose success relies on working with groups homogeneous in age, as well as the desire to reduce fighting behaviour and the associated stress (“***Mixing has the ultimate negative impact […] they do fight and they can cause quite a bit of injury***”). This suggests that the welfare of pigs was considered in the decision-making rationale for this management procedure, although not explicitly stated.

#### Mixed or split sex groups

Maintaining single sex groups is a management procedure to reduce sexual and aggressive behaviours that can occur between males and females, and to tailor feeding programs for gilts and boars. Three respondents (30%; *P* = 2, V = 1) indicated that they maintained mixed sex groups, four veterinarians (40%) worked with clients who either mix or split sex groups, and 30% (*P* = 2, V = 1) always split sex groups. Those who pen pigs in mixed sex groups explained that they do not see a reason for splitting sex groups, with one veterinarian acknowledging that they have never been able to manage split sex groups to capture the full value of this practice. Those who opted to split sex groups tended to have specific reasons for their decision. For example, two veterinarians stated that managing split sex groups is more common in barns that immunocastrate pigs, allowing diets to be tailored for boars and gilts separately, however, improving pig welfare was not a driver. While some observed faster growing barrows because of this strategy, others did not. Welfare was indirectly mentioned by those opting against splitting of sex groups; one producer (immunocastration user) and one veterinarian (representing clients that occasionally immunocastrate) expressed a belief that splitting sex groups is detrimental to the aggression levels among pigs (“*I suspect that with the mixing of sexes it probably calms things down if anything*”).

#### Resource and space allocation

Most respondents (90%) indicated that their resource allocation management strategy was based on industry (i.e.: the Code of Practice for the Care and Handling of Pigs) or manufacturer recommendations for the equipment on-farm, i.e.: specific feeder technology used, and all respondents indicated compliance with the minimum industry space requirements. Some noted that they may exceed recommendations to ensure that dominant animals cannot monopolize all feeders/drinkers, to improve access of subordinate animals and facilitate better growth rates. While this suggests that productivity and growth targets were the main drivers for the decision to exceed resource allocation recommendations, respondents also noted that it can alleviate aggression (“***If you’re really limited on water access […] then that can impact welfare […] pigs will have to fight to […] drink***”). Seven respondents (70%) were aware of the negative effects of overstocking on animal welfare and productivity. An increased likelihood of tail and flank biting, leading to a higher infection and mortality risk were among the negative consequences listed by participants. Respondents also indicated that pigs were more likely to experience stress, and less likely to have adequate access to food, resulting in a slower growth rate (“*If you pack them too tight then you definitely see negative behaviour […]. You’ll see more animals that are falling out and not getting enough feed*”). One producer questioned the push for higher space allowances by initiatives such as Prop 12 in the U.S., indicating that offering additional space alone is not enough, highlighting the need to consider the quality and functionality of the space offered (“*More space is not necessarily good space*”). Moreover, respondents noted that factors such as pen layout, floor quality, and environmental management also play a crucial role in ensuring good welfare when managing pigs in social groups.

#### Environmental enrichment

Environmental enrichment is provided to pigs to encourage expression of natural behaviours, reduce boredom and stress, and improve overall welfare [28]. Eight (80%) respondents indicated that they provide enrichment to their pigs, and mentioned the use of balls, PVC pipes, ropes, chains, commercially available items often termed as “toys”, salt blocks, and music. When asked about the rationale for providing enrichment, one producer cited increasing regulatory pressure in the form of requirements in the Code of Practice for the Care and Handling of Pigs for the incorporation of enrichment (“*Canadian Pork Excellence has been really pushing environmental enrichment*”). However, the benefits of enrichment to pig welfare were also recognized and seemed to contribute to its use. Two producers stated that enrichment is used as a way of preventing or managing tail biting among pigs, as engagement with enrichment helps to keep pigs occupied, decreases fighting, and facilitates social play. One veterinarian reflected that strategic use of enrichment is key to ensuring its effectiveness in improving welfare (“***Enrichment done well has a positive influence on welfare***”) and used the example of rotating enrichment items to maintain novelty.

Nevertheless, two respondents (20%; *P* = 1, V = 1) who provide enrichment for their pigs voiced frustration over the cost of enrichment that is destructible and therefore more frequently used by pigs (“*We have had some sow farms that have purchased those pig toys, and it was an absolute waste of money because the sows ate them*”; “***I think the cost limitation is frustrating***”), a feeling which may be exacerbated by the lack of observed benefits of enrichment to productivity metrics (i.e.: feed conversion rates: “*I wouldn’t say that we’ve seen an improvement in production metrics as a result of enrichment*”), and thus no obvious return on investment. Only two respondents (20%; both American) indicated that they do not provide enrichment to their pigs (*P* = 1, V = 1 representing clients who do not provide enrichment). A lack of requirement, guidance, and doubts regarding its effectiveness in improving pig welfare were cited by the producer respondent as reasons for not providing enrichment to pigs (“*I’ve got very big hesitation to do something that hasn’t been agreed on or hasn’t been asked of us to do. Personally, I would really like to be able to see data and research regarding how enrichment affects, and contributes to the pig’s welfare*”).

### Interrelationships between management procedures, staff well-being, and business sustainability

After discussing specific management procedures and the rationale behind decision-making, respondents were asked to reflect on how these procedures and animal welfare affect staff well-being. They were also invited to reflect on how this may affect pork production and their operations’ sustainability goals, particularly in relation to economic viability and profitability, efficiency, and ethically and environmentally responsible practices.

#### Management procedures and staff-wellbeing

Management procedures associated with pain, injury, and disease in pigs were reported to have a negative impact on staff well-being by nine (90%) respondents. Six (60%) respondents indicated that staff disliked piglet processing procedures involving the routine removal of body parts (i.e.: surgical castration and tail docking), describing it as “a task no one enjoys doing”. Five respondents (50%) who had switched from surgical castration to immunocastration emphasized that they had no desire to return to surgical castration, largely because of the positive impact on staff (“*as soon as we tell [the staff] they’re not physically castrating anymore, they are thrilled*”), however, a specific reason for this was not provided. One producer who uses pain control during processing explained that new staff often find the procedures distressing, but become desensitized over time (“*It can be quite traumatizing to people initially […] but you get used to it”*). This suggests that staff are inherently uncomfortable with invasive procedures, regardless of whether pain control is used or not. Moreover, their initial distress reflects a level of empathy towards the pig, and recognition of the ethical conflict involved. Repeated exposure normalizes these procedures, leading to desensitization, likely as a self-protective response to facilitate task completion, indicating a negative psychological impact on staff. Moreover, two veterinarians and one producer described the emotional and physical toll of a sick herd on staff (“***People get really frustrated when their pigs are sick***”; “*[…] Healthy herd, happy staff*”). While pig distress was not explicitly linked to staff empathy for the animals, it was evident that it elicited emotional responses; three veterinarians and one producer indicated that staff feel helpless during disease and tail biting outbreaks (“***I have this clear memory of this producer who just walked outside and started crying [because of a tail biting outbreak***]”). Two veterinarians highlighted that repetitive piglet-handling tasks, such as surgical castration and tail docking, can cause both physical injuries and mental strain for staff (“***Any job [referring to surgical castration and tail docking] that you do repetitively, do physically, you can have injuries, but also mentally. I mean, none of us are made to do the same eight hours job***”). In this case, the mental strain likely stemmed from the physical demands and repetitiveness of piglet processing, rather than from the emotional burden of carrying out a painful procedure. One producer noted that lifting piglets for processing could improve piglet welfare by increasing their comfort with staff handling, but also acknowledged the physical burden this placed on workers. Producers similarly described castration as labour-intensive and a source of job dissatisfaction, especially when staffing levels were inadequate (“*If you do have to castrate you are better off making sure that you are scheduling the right amount of people to do the right amount of labour so that you don’t start wearing people down […] Castrating and tail-docking is definitely labour intensive*”). In contrast, immunocastration was described as making staff happier because it reduced both workload and physical strain, suggesting that the shift to this practice was driven more by benefits to staff efficiency, physical health, and morale than by direct concern for piglet welfare.

Seven (70%) respondents identified euthanasia of sick or injured pigs as a significant challenge for staff well-being. Two respondents described it as conflicting with the perceived role of staff as animal caretakers (*“It’s hard to euthanize a sow… when what you’re really there to do is take care of the healthy animals*”), one respondent noted that access to euthanasia methods that are perceived as more humane by the staff can play a role in ensuring that timely euthanasia is provided (“***[…] blunt force trauma is still being used for piglets. They are effective tools, but they are not so human friendly***”), and one attributed the reluctance to perform euthanasia to a limited understanding of welfare outcomes, including the importance of timely euthanasia to reduce prolonged suffering to support animal welfare (*“Once you explain to them that [the pig] is going to get its butt kicked its entire lifespan… then it becomes a lot easier to make that decision*”). This aligns with the consensus among respondents (80%) that staff training and support are essential for positive staff well-being. High staff turnover can result in staff lacking the extent of training required to develop proficient skills and confidence to handle animals properly, recognize health and behaviour issues, or understand the rationale behind management decisions, which in turn can place a greater psychological burden associated with decision-making around procedures like euthanasia (“***If people feel like they haven’t gone through sufficient training, you can see how much it stresses them, especially when they’re doing surgical procedures***”).

Respondents agreed that overcrowding impacts the pigs, staff, and productivity metrics alike. It was noted that overcrowding makes it challenging for the staff to perform health checks safely, directly impacting the pig’s welfare through missed treatments or delayed euthanasia. Two veterinarians connected pig discomfort resulting from overcrowding with a negative impact on staff well-being and their ability to connect with the animals (“***Pigs that are uncomfortable and crowded and impossible to walk through have a negative impact on the staff and often has a negative impact on the staff’s ability to connect with the animals***”). Two veterinarians noted that practices which improve pig welfare, positively impact staff well-being and productivity, i.e.: providing pigs with enrichment and allowing pigs more space to better facilitate a connection between the staff and animals (“***In farms where they have a relationship or a connection with the animal, we see more positive production and welfare outcomes***”). Moreover, eight (80%) respondents cited negative impacts on production and profit, because of reduced reproductive capacity, tail biting, and poor feed conversion and average daily gain resulting from overcrowding.

#### Challenges to business sustainability

Respondents consistently emphasized that broader labour shortages exacerbate the above challenges, affecting both staff well-being and animal welfare. Understaffing leaves employees overworked, stressed, and dissatisfied (“***Low staffing numbers can create an instance of being overwhelmed***”), and producers noted that lack of vacation time and high turnover further reduce job satisfaction and make staff retention challenging. Labour shortages directly and indirectly compromise animal welfare by limiting the time staff can devote to essential care tasks, such as walking pens, monitoring health, and performing husbandry procedures. Two veterinarians described how inadequate staffing leads to rushed work, missed treatments, and emotional strain for employees who know they cannot give pigs the attention they need (“***[…] I see staffing issues really start to stress people from both the productivity but also from an animal care perspective. You really start to see the anxiety go up***”).

Respondents also recognized that poor animal welfare negatively affects staff well-being, particularly in relation to painful procedures, euthanasia, overcrowding, and labour shortages. However, they did not make the reverse connection, linking poor staff well-being to reduced productivity or broader business sustainability. In addition, no respondents identified any implications of these factors for environmental outcomes such as emissions. Some respondents did, however, demonstrate an understanding that maintaining social licence is important for business sustainability (“***Because we sell meat, we want people to understand what we’re doing and buy our meat and feel that we’re taking good care of our pigs***”). When asked about the interrelationships between animal welfare, productivity, staff well-being and business sustainability, it was clear that respondents connected indicators of poor animal welfare to poorer performance and productivity metrics that are key determinants of economic profitability (i.e.: feed conversion, average daily gain, pigs per sow/year, body condition, injury scores, mortality), and therefore to overall sustainability of the business model (“***Animal welfare, barn management, individual pig management, performance. They’re all very much interrelated***”; “*When you’re not providing your animals with everything that they need, your production will suffer*”). For instance, in discussions around productivity, metrics described as tools to monitor welfare often included metrics that are key determinants of economic profitability (“*I guess you could also probably include metrics like body condition and injury scores*”; “*Feed conversion and average daily gain*”). This highlights the acknowledgement of the links between welfare and productivity, even when this was not always explicitly stated by the respondents. Six (60%) respondents indicated that injured, stressed, or sick pigs negatively impact production and profit as these can lead to tail biting, which may then increase wounds, infections, and lameness. Mortality was discussed as a universal indicator of welfare and productivity which could be understood by different stakeholders [“***(…) if you have high mortality, (…) it comes back to welfare. (…) it’s the same KPI and the same results***)”. Eight respondents (80%) indicated that healthy, content pigs result in improved productivity and profit, and metrics such as average daily gain, feed conversion ratio, and meat quality were cited by respondents in this discussion (“***Average daily gain, the body condition score. Making sure they’re healthy, have adequate food and water and the nutritionists are doing what they need to be doing. So that’s going to affect feed conversion***”). Moreover, such metrics were linked to broader sustainability goals (“***Feed conversion ultimately is the number one because feed is 70% of their cost in the growing pig. If they can get the same growth with less feed, then it’s going to save money. Average daily gain too because space cost something as well. Mortality of course is very costly, if you lose an animal that you put a lot of money into it’s very costly***”). Furthermore, the frequent reference to animal welfare monitoring metrics in discussions of productivity and sustainability goals indicates a clear appreciation of the role of good welfare in achieving sustainability (“*Anything that improves your efficiency, is good for sustainability. If we can use ten pounds of feed less to get the same weight gain on a pig, well that’s ten pounds of feed that didn’t need to be transported all over the place, didn’t have to be grown or could be used for something else*”).

Despite acknowledging the positive effects of good welfare on productivity and sustainability, 30% of respondents reflected on competing interests between welfare and profitability (“***There is a point where if you look at farm and its profitability is only $3 per pig, as soon as you start spending beyond your available cash, the farm is no longer viable and that’s the end of the conversation. There is always competing interest for that margin. Without the margin the farm cannot be operational”;*** “*There is always more to do, and it always costs money, but it doesn’t always translate into better returns*”). These competing views are presented in Table 3. Respondents felt constant pressure to improve welfare, yet often did not observe a corresponding improvement or return on investment. One respondent noted that pain medication is difficult to justify without direct financial return. Limited understanding of welfare criteria (e.g.: 5Fs) likely amplifies the perceived scale of the task of improving welfare, contributing to frustration and misconceptions about its relationship to production performance. One veterinarian emphasized that producers tend to adopt welfare-improving strategies primarily when there is an economic benefit. This is problematic because many welfare improvements, such as reduced stocking density do not bring clear or immediate financial gains. For example, reducing stocking density can improve pig welfare, but producers may see they can immediately obtain greater economic return from higher pig throughput, without evaluating the less obvious economic impacts of overcrowding on production costs in the longer-term, including herd health issues resulting from more pigs per air space, increased stress and compromised immune function, reduced growth rates, and higher mortality. One veterinarian emphasised that science is essential to resolve perceived contradictions between welfare and production, and to highlight productivity benefits when promoting welfare strategies.

**Table 3:** Stakeholder perceptions on the relationship between animal welfare, productivity metrics, and business sustainability

Factors	Welfare benefit	Economic benefit	Welfare drawback	Economic drawback
Space allocation	No response provided	Overstocking can result in increased production and profit (*P* = 3)	Overstocking negatively impacts pigs (*P* = 3, V = 3)	• Overstocking will increase mortality and decrease profits (*P* = 1, V = 1) • Overstocking may impact reproductive ability as pigs’ needs are not being met (*P* = 1) • Overstocking may improve feed conversion but reduce average daily gain, meaning pigs take longer to reach market weight (V = 2)
Resource allocation	No response provided	Cheap feed saves costs up front (*P* = 1)	Cheap feed causes health issues in pigs (*P* = 2)	• Health issues from cheap feed ultimately end up costing more (*P* = 1) • Limited feeders and drinkers slow growth and increase time in the barn as pigs take longer to reach market weight (*P* = 1, V = 1)
Staff knowledge and interaction	Knowledgeable staff identify welfare issues earlier (*P* = 1)	Staff interaction with pigs improves quality of meat (V = 1)	No response provided	No response provided
Production indicators	No response provided	• Pigs per sow per year related to efficiencies (*P* = 2) • Feed conversion and average daily gain related to efficiencies (*P* = 2, V = 3) • Body condition related to reduced costs (*P* = 1, V = 1)	No response provided	• Injury scores and mortality related to lost profit (*P* = 1, V = 2) • Cost of the facilities, feed, water, and animal health treatments decrease profit (*P* = 1, V = 1) • Low average daily gain means having to sell pigs at a lighter weight (V = 1)
Tail biting or sickness	No response provided	No response provided	No response provided	• Tail biting wounds, infection risk, and lameness (*P* = 1, V = 1) • Disease outbreak increases culls and mortality (*P* = 1, V = 1) • Disease or infection increases treatment costs (V = 2) • Stress reduces meat quality (*P* = 1) Sick pigs grow slower (V = 1)
Other	No response provided	General animal welfare related to improved production/profit (*P* = 4, V = 4)	No response provided	No response provided

P - Producer; V - Veterinarian responses

## Discussion

With animal care as a major pillar of the pork business sustainability model, this study aimed to understand pork industry stakeholders’ perceptions of animal welfare, its role in the management decisions they make for their animals and in supporting sustainable production. This was achieved by assessing how swine producers and veterinarians define animal welfare and the metrics they use to monitor welfare on-farm; stakeholder understanding on how their farm management procedures influence welfare, and an assessment on whether animal management decisions were made based on welfare or productivity, or whether barriers to implementation exist; as well as stakeholder understanding of the relationships between animal welfare and broader pork production sustainability metrics. Insights from this work can be used to promote greater understanding of welfare within the pork business sustainability models, and could be framed within the context of the 5Fs to support understanding. While the scientific community increasingly recognizes the importance of animal welfare to both productivity and sustainability in animal production [8, 9], this awareness is less evident within the pork industry, potentially undermining the sector’s long-term viability [2], but also likely highlighting concern for competing interests between welfare goals and tight profit margins.

### Stakeholder understanding of animal welfare

Responses indicated that stakeholders consider animal welfare important, but do not think about welfare in full alignment with existing welfare assessment frameworks such as the 5Fs. For example, respondents focused more on criteria such as provision of sufficient food and water than those related to fear, distress and the freedom to perform natural behaviours. When asked about what animal welfare means to them, respondents interpreted welfare primarily in terms of physical, visible states (i.e.: injury to the pig’s, body condition score), with many stakeholders overlooking behavioural indicators that can reflect poor psychological welfare. However, injuries on pigs could also reflect behaviours in the pigs (i.e.: tail biting), and this was acknowledged. Similar perceptions were reported among Canadian swine industry stakeholders by Spooner et al. (2014), and it is noteworthy that this view persists over a decade later. Albernaz-Gonçalves et al. (2021) observed the same trend among Brazilian swine industry stakeholders, where definitions of welfare centred largely on physical aspects. Taken together, these findings suggest that a focus on physical, outwardly visible states of welfare (i.e.: sick pigs, lesions on the body) may represent a global trend for indicators the pork industry uses to monitor welfare. Behavioural indicators of welfare were reported by respondents in the current study, but it was veterinarians that were more likely to reference these measures, as well as elements of animal welfare encompassed by the 5Fs, potentially reflecting their formal training in animal health, behaviour, and welfare; training that appears to be limited among producers/barn staff. This points to insufficient staff education on core principles of animal welfare and pig behaviour and can explain why the 5Fs framework is not commonly fully applied in practice. It may also indicate that, despite its simplicity, the 5Fs framework remains too unachievable for staff to consistently implement on farm, especially when the emphasis is on freedom, in a production system that can put forward many constraints for animal management. Limitations among stakeholders to understanding all areas that apply to welfare may partly explain the reluctance to discuss welfare standards and strategies on-farm [29, 30]. This reluctance was evident in the challenges encountered when recruiting stakeholders for interviews, suggesting a broader discomfort with discussing welfare. The same reluctance is mirrored in the tendency for only positive narratives to be portrayed in the media [31], underscoring the need for more transparent and critical engagement on animal welfare in the pork sector.

Respondents acknowledged the links between management procedures used in their systems (i.e.: castration, tail docking, and space or resource allocation strategies) and their positive or negative impacts on pig welfare. When asked about the rationale behind these procedures, respondents primarily emphasized productivity gains and economic considerations, rather than welfare, indicating that economic drivers often take precedence over animal welfare in decision-making. This is not unexpected, for it is of central importance that a business remains economically viable, and with the pork sector having tight margins, volatile pork prices, facing rising production costs, and inadequate compensation for pork produce, this creates persistent financial pressures [2, 3]. Economic viability underpins every aspect of pork production; without it, there are no resources to pay staff, care for animals, or implement welfare strategies [1]. Because welfare improvements typically equate to additional costs, initiatives that do not yield immediate profitability are often perceived as an imposition that conflicts with business viability [17]. Moreover, producers tend to overlook the long-term benefits of animal welfare for profitability and its contribution to the sector’s overall sustainability, which further hinders the uptake of welfare strategies [17]. This may be further fuelled by the majority of global pork markets not requiring compliance with specific animal welfare standards [32]. Economic analysis and pork life cycle assessments incorporating animal welfare strategies would be valuable to help the pork industry evaluate the long-term strategies for meeting economic and market sustainability requirements.

The interview responses highlighted a disconnect between the stakeholders’ perceptions of what should be done to uphold welfare standards, and what is done in practice as reflected by the management procedures discussed during the interviews. Namely, some producers noted that animals are productive when their needs are fully met. Yet, despite this acknowledgement, meeting animal needs through addressing pig welfare requirements was not a key driver in many management decisions, and there were many misconceptions about how certain management procedures affect welfare. For example, some respondents perceived environmental enrichment as having little value. One respondent from a surgical castration farm claimed that castration does not negatively affect piglets. Since surgical castration is widely recognized as painful [33], such responses raise questions about whether they reflect genuine beliefs or serve as a psychological rationalisation to justify the continuation of a painful procedure. If the latter, this signals a degree of dissociation and cognitive dissonance in regard to surgical castration. Similar justifications appeared in responses regarding tail docking. Some producers communicated that picking up piglets for docking was beneficial because it provided staff with an opportunity to inspect piglets closely and habituate them to human handling, thereby reducing stress and fear. However, this reasoning can be seen as flawed. Piglets are already handled for iron injections, and therefore no additional opportunity for handling and inspection should be needed. Moreover, handling associated with painful procedures is more likely to increase the pig’s fear of humans than reduce it [34]. Again, such explanations may reflect attempts to rationalise the continuation of painful practices, though they could also indicate genuine, but misguided beliefs. In either case, they point to a lack of understanding of pig behaviour, biology, and how pigs respond to stressful stimuli, which further highlights deficiencies in staff training.

The responses also revealed evidence that businesses did not incorporate welfare management strategies that required additional costs or labour (i.e.: pain control, enrichment) unless there was a requirement to, either from the market or Codes of Practice (i.e.: NFACC, 2014) At the same time, there was evidence that industry requirements (i.e.: national industry requirements and guidelines set by the pork industry) can educate stakeholders on appropriate animal care. For example, Canadian respondents providing pain control at piglet processing stated this was to comply with the Code of Practice requirements, and there was acknowledgement that this was painful and that ceasing physical castration through adoption of immunocastration removed the need for pain control for this procedure. However, a respondent who did not provide enrichment stated they had received no guidance on the subject and would like to see data demonstrating its benefits. Extensive evidence on the importance of enrichment for pigs exists [35–37] and is readily accessible. This points to an industry-level failure or avoidance to disseminate knowledge on certain welfare topics, potentially to control the rate of financial investment into welfare strategies required by producers. A similar conclusion was drawn by van de Weerd and Ison (2019), who also identified inadequate industry knowledge dissemination as a barrier to the adoption of welfare strategies. There was also some evidence of unwillingness to adopt practices that could improve welfare. For example, one respondent reported not having seen tail biting in their herd for five years, yet still continued to tail dock. In such cases, the absence of tail biting should encourage a shift away from docking, but no such change had occurred, suggesting there is comfort in perpetuating existing routine practices over exploring ways to reduce routine painful procedures for pigs. Together, these examples highlight the disconnect between how pork industry stakeholders perceive and talk about welfare, and how it is implemented in practice, as well as the limited initiative to improve welfare in the absence of formal industry or legislative requirements.

Consideration for welfare emerged in the stakeholders’ perspectives, but it was often not explicitly acknowledged as a welfare consideration. Respondents often described management practices such as treating compromised or sick pigs as important, without explaining the reasons behind them. This may be because they assumed it was self-evident that such actions in the context of sick/compromised animals were motivated by welfare concerns rather than solely by productivity goals. The overlap between the metrics used to monitor welfare (i.e.: number of treated pigs, mortality, body condition score) and key indicators of productivity (i.e.: low mortality) further suggests that producers recognise to some extent, the connections between welfare, productivity, and sustainability, even if these are not consistently translated into practice. For instance, although good welfare was acknowledged as important, some respondents simultaneously perceived certain requirements as a trade-off with profitability, leading to the persistence of management practices that may compromise welfare. Such perceptions were also described for Canadian swine industry stakeholders by Spooner et al. (2014), and globally by Niemi [38].

A recognised challenge for pork business sustainability is labour availability. Therefore, efforts to make the working environment more attractive for employees is key to supporting improved labour retention. As also reported by others [39], respondents acknowledged the negative impacts of poor animal welfare on staff. Repetitive tasks such as castration and tail docking were described as mentally taxing, though it was unclear whether this was due to the monotony and the physically demanding nature of such work, or the mental strain of repeatedly performing painful procedures on animals. Responses also revealed empathy towards the pigs, such as one vet recalling a producer crying during a tail biting outbreak, and vets reporting that staff have a hard time when there is sickness in the herd. Thus, it indicates that when pain (including routine painful procedures), injury and disease are present in the barn, it has negative impacts on staff well-being, and efforts to reduce these impacts are rewarding for staff. From the responses, it remains uncertain whether this reflected genuine compassion for the animals or stemmed from other personal or situational conflicts, such as the impact on business production goals and time constraints, although it is appreciated the emotions could stem from a combination of these. Veterinarians also noted that on-farm training in key procedures such as castration and euthanasia of compromised animals was inadequate; a gap that also negatively affected staff well-being.

It should be acknowledged that in some instances, stakeholder perspectives were difficult to interpret. Differences in educational and cultural backgrounds between pork industry stakeholders and researchers may have shaped or constrained how these perspectives were understood. As a result, some important connections appear to remain unmade. For instance, while stakeholders recognized that poor animal welfare negatively affects both staff well-being and productivity, they did not explicitly link poor staff well-being itself to reduced productivity or broader business sustainability. Improved feed efficiency, growth rate, reduced veterinary treatments and mortality were all metrics of the production efficiency of the operation that could positively influence economic return and contribute to environmental sustainability. Thus, where management practices that enhance animal welfare can support this (i.e.: timely vet treatments, prompt euthanasia), it was clear it supported the sustainability model. However, if animal welfare enhancing activities required investment and could not produce a productive return, such as was commented on for enrichment, there was question over the value of the practice. Notably, stakeholders did acknowledge the importance of good animal welfare for maintaining social licence as being integral to business sustainability, and this was more widely recognised by respondents than requirements for environmental sustainability. This is somewhat surprising, given that societal pressure for environmental sustainability in agriculture has been established for much longer than societal pressures relating to animal welfare [40]. However, this likely reflects lower pressures for environmental requirements in North America. Clearer expectations for animal welfare standards, combined with economic analyses and life cycle assessments of such implementations are required for the industry to evaluate sustainable strategies.

## Conclusions

This study demonstrates that while the importance of animal welfare to sustainable pork production is recognised, it is not yet fully understood or integrated in the workplace culture or business models of pork production systems. Although producers did not articulate a clear definition of welfare, they nevertheless referred to welfare indicators – primarily visible, physical measures rather than behavioural ones, suggesting that welfare considerations are present, and the indicators used largely overlap with indicators that have economic consequences for production. Stakeholders acknowledged the value of good welfare for both profitability and staff well-being, yet it was clear that welfare often came second to economic gains, and practices that incurred an upfront cost were not adopted unless required. This tendency may reflect a limited appreciation of the broader long-term benefits of welfare for economic viability and sustainability, and also the delicate financial risk management the industry must balance in a volatile pork market. A key recommendation is that the pork industry move away from viewing welfare as an externally imposed requirement and instead embrace it as a framework for meeting animals’ needs and enhancing long-term business sustainability. Increased education to enhance stakeholder understanding of welfare could help to improve engagement and adoption of animal welfare strategies. Progress towards this shift could also be supported by research studies that not only investigate welfare improvement strategies and practical welfare assessment tools, but also incorporate economic analyses of those strategies, combined with life cycle assessments. Demonstrating how welfare initiatives can be integrated into production systems to enhance productivity could help stakeholders better recognize and appreciate the role of pig welfare in the overall sustainability of the pork production model, and for the industry to navigate meeting animal welfare expectations in a more sustainable model.

## Electronic supplementary material

Below is the link to the electronic supplementary material.

Supplementary Material 1

## Data Availability

The datasets generated and/or analysed during the current study are not publicly available due the potential for identification of individual responses, but are available from the corresponding author on reasonable request.
