# Supplementary material for: Animal welfare and sustainability in pork production: a pilot study of farmer and veterinarian perspectives in North America
Source: Porcine Health Manag. 2026 May 30;12:43. doi: 10.1186/s40813-026-00521-5 (PMC13435494; doi:10.1186/s40813-026-00521-5)
Supplement: Supplementary file 1 [file 40813_2026_521_MOESM1_ESM.docx]

**Interview of Pork Producers and Swine Industry vets – Script**

Consent form:

Hi, [insert name]. I’m going to talk a lot at first and then not much after. The objective of this interview is to identify what parameters are important for animal welfare and business sustainability for pork producers. Your participation in this work will support the development of a tool to help pork producers make managerial decisions that can better meet animal welfare and sustainability targets. This tool aims to increase animal welfare, profitability, and sustainability of the swine industry. Before we start, I think it is important we discuss your rights and the objective surrounding this interview.

Firstly, this interview will be recorded and transcribed word for word. During the interview, you may request that the record is turned off at any time during the interview, without giving a reason. Once the interview is completed and a transcript of the interview is made, you will have the opportunity to review the transcript for a period of one week to add or remove information as you see fit. If there are no changes after this week, the interview will be used as is. Your data, audio recording, and transcription of the recording will be stored on a secure research server at the University of Saskatchewan. Only approved personnel will have access to these recordings. In turn, participants agree to not make any unauthorized recordings of the content of the data collection session.

Secondly, your participation is voluntary, and you reserve the right to answer, or not answer, the questions that are asked of you. You may withdraw from the project at any time and for any reason and your participation will in no way affect your position/employment or how you will be treated. If you do choose to withdraw, please provide a written request to the PI, Dr. Seddon, and your data will be deleted from the research project and destroyed. You will receive a notification when the information pertaining to your participation is destroyed. Your right to withdraw from the study will apply until one month after the date of the interview. After this point, some form of data analysis may have already occurred, and it may not be possible to withdraw your data. If you choose not to withdraw, your data will be stored for up to five years post-publication, after which the data will be destroyed or deleted beyond recovery.

Lastly, this interview is scheduled to take an hour and a half. This interview is designed to ask you questions regarding what is important to you, from an animal caretaker and business perspective, for the welfare of the animals and meeting the sustainability targets of the business. I want to stress that I am here not to judge or criticize your responses, we are genuinely interested in hearing what you have to say. I will interject only when I need to for the sake of time, other than that the floor is yours to respond as you see fit. If a question feels repetitive, feel free to respond by saying that you’ve answered this previously. This information will be published in a scientific journal, but your identity, at all times, will remain confidential.

With that all being said, do you have any questions?

Do you consent to this interview?

Introduction:

Thank you for agreeing to take part in this interview. I think it is best to start the interview with your background and demographic information.

Interview:

Date:	Interview Number:

Location:

# i. Background/Demographics

1. Briefly, can you please tell me your current position?

2. How many years have you been in this role?

a) How many years have you been working with pigs?
   a. Under 5 years
   b. 5-10 years
   c. 10-15 years
   d. Over 15 years

3. Is there a stage of pork production that you specialize in (i.e., farrowing, nursery, grower/finisher, or a farrow-finish)?

4. What professional education have you completed?

Thank you for your answers. Now I would like to ask about your work experience.

# ii. Work experience

1. Have you always worked with pigs?

*Probes:*

- *What other animals have you looked after?*
- *Did you look after these animals in the same capacity?*

2. Are you responsible for management decisions? If not, what role is responsible for these decisions.

3. Are you responsible for meeting the animal welfare and sustainability targets for your employer?

# *Probe/Definition of animal welfare: The physical and mental state of an animal in relation to the conditions in which it lives and dies i.e., how an animal is coping with the conditions in which it lives.*

Thank you for that overview. Now I would like to focus on animal welfare in pig farming. For these upcoming questions, I would like for you to draw from your personal experience of how husbandry influences animal welfare and what you have observed on farm.

# iii. Animal welfare and the importance of animal welfare indicators

1. We recognize that the understanding of animal welfare can be shaped by individual experiences. When you hear the term animal welfare, what does that mean to you?

*Probe/Definition of animal welfare: The physical and mental state of an animal in relation to the conditions in which it lives and dies i.e., how an animal is coping with the conditions in which it lives.*

* This can be a question where they need time to formulate an answer. I can also see some people going off on a separate, but tangentially related, thought of what animal welfare is doing to them, but not answering the main question of what is the meaning of the term to them (i.e., what do you think is important to consider when caring for an animal).

2. Are there components of animal welfare that you feel are more important than others? If so, can you tell me a little bit about them?

* This can be a question where they need time to formulate an answer.

3. Can you briefly tell me what indicators you use to monitor animal welfare on farm?
A. Why did you/the business decide on these indicators?

4. Relating to animal wellbeing/welfare and the quality of life experienced by an animal, how do you consider these indicators affect the pig itself?

# Could you reflect on how the indicators that you mentioned affect the pig’s welfare?

# *Probes: Fewer animals displaying skin lesions (less aggression in the group, less emotional upset), less incidents of tail biting (lower stress in the group, pigs’ needs are being met), less use of antibiotics (animals are experiencing better health, lower stress), etc.*

# *Probe if needed: For the purpose of this work the quality of a pig’s life refers to the wellbeing of an individual over an extended period of time and considers the balance of positive and negative states experienced by an individual, leading to whether an individual experiences a life of predominantly good welfare, or predominantly poor welfare. Quality of life considers the emotional experience of the animal with regards to positive and negative experiences.*

* This can be a question where they need time to formulate an answer. There can be a lot of variation in the answers depending on what the answer in 3.

Thank you for that information. Now I would like to move on to talk about the management of a growing herd. You can draw on anything you feel might be important for us to talk about in these stages. Is this alright with you? As a reminder, you do not have to answer any questions you wish not to.

# iv. Management of animals and its relationship to animal welfare and production performance.

1. Can you provide us with a brief overview of the management practices that are in place for the growing animals? If we start with piglets in the farrowing house [If individual does not manage piglets, start with the stage they manage, i.e., nursery, grower, finisher].

Can you provide us with a brief overview of the management practices that are in place for piglet processing?

*Probes: What about management at processing? Tail docking, castration, teeth clipping, weaning practices? Enrichment? Improvest use and what changes in production (including health performance) has been observed? Decision to raise entire male pigs?*

a. What is the reasoning for these management decisions? [Question may need to be repeated for each management decision provided leading the conversation through: i.e., processing, weaning practices, provision, or lack of enrichment etc.].
b. How do you feel, based on your observations, that this management/these procedures impact the welfare of pigs? [Please elaborate on what you observe in the pigs to inform your opinion].
c. Have you considered using Improvest?
d. If not mentioned in the responses: do you use pain control for processing? Why/why not?
e. Do you/your barn reference any guidelines or protocols when you are considering whether to use pain control for physical castration or immunocastration?

2. If we now move to management of the growing animals:
   a. Can you tell me about management after weaning?
   b. Have you chosen to use split sex groups?
   c. If yes, do you consider there are specific challenges or opportunities with the management of these animals in the spilt sex groups?

*Probe: split sex for better vaccination management, optimising feeding.*

d. Do you try to maintain animals in the same group throughout growing? Or do you have to mix groups later in the growing phase? [regrouping resulting in aggression].
e. How do you feel, based on your observations, that this [weaning and grouping practices] impacts animal welfare?

*Probes: behaviour of the animals, health challenges, aggression, weight loss and performance.*

3. What is your guidance for determining the space allowance provision for the growing herd?
4. Do you consider space allowance to be something that is easy to accommodate?
   a. Why or why not?
   b. What have you observed with regards to how space allowance impacts the welfare of the pig?

*Probes: Change in behaviour, visibly no space for all to lie down, agitated animals, reduced growth, health challenges.*

5. What is your approach for resource allocation in the pen? [Expect information on drinker and feeder provision, lying space etc]
   a. Based on your experience, how does resource allocation influence animal welfare?

*Think about: ensuring pigs have easy access to food and water and that all animals have equal access. Do pigs have enough space allocation to prevent overcrowding. Think of enrichment and mental stimulation.*

b. Linking to above, and performance?

*Think about: pigs not having sufficient access to the feeder would reduce growth, overcrowding can also increase stress decreasing growth.*

* Both of these questions could take some time to formulate an answer.

6. Is environmental enrichment considered for your indoor system? Why or why not?

*Probe/Definition environmental enrichment: Provision of substrate or specific objects that pigs can root and chew on – providing an outlet for pig specific behaviours.*

a. How do you feel the provision (or lack of) influences animal welfare? Probes*: What have you observed in pigs with/without enrichment? [May encounter individuals who have never observed pigs with enrichment].*

7. Do you experience tail biting in the growing herd?
   a. How do you manage an outbreak?
   b. What do you consider a tail-biting outbreak is telling us about the welfare of the pigs at the time of an outbreak?
   c. What husbandry management factors have you identified as being linked to an increase in tail-biting?
   d. What changes in the animal do you observe before a tail biting outbreak?
   e. How does tail biting impact the productivity of the pigs and productivity targets?
8. How does your barn manage sick animals?
   a. [If not mentioned:] Do you use hospital pens?
9. Finally: Is there anything else you would like to tell us about management decisions for the animals and how it influences their welfare from what you have observed and the performance?

Thank you for all that information. We only have two sections left. If it’s alright, I would like to change our focus now on your experiences with how managing animals and their welfare impacts staff wellbeing.

**v. Influence of animal management on staff wellbeing**

1. With regards to animal management and their welfare, from your experience, what do you see as the most challenging issue for staff wellbeing?

[If not already brought up], if we can go back to piglets, how do you think performing (tail docking, castration, etc.) procedures impacts yourself and staff wellbeing?

1. How do you think piglet processing (tail docking, castration etc.) impact staff wellbeing?

*Probes: Staff moral and mental wellbeing during painful procedures*

*Staff physical wellbeing based on labour outputs (extra work created by processing).*

a. [If these have been raised as a challenge in previous conversation ask about how specific management decisions may impact staff i.e.] how does a lack of space allowance influence staff wellbeing?

# 2. Do you have any recommendations as to what could be done to help reduce the impact of animal management on staff wellbeing? Please be specific with regards to the approach for which management.

*Probe: Or, to address these issues as an industry?*

3. I know this was a very brief discussion of worker wellbeing, but is there anything relating to worker wellbeing that you think is important for us to recognize?

Thank you so much for all that information. In this final section, what I would like cover focuses on productivity measures. As always, you can draw on anything you feel might be important for us to talk about the interrelationship between productivity and welfare. Does this sound good?

# VI. Productivity Measures and their interrelationships with other measures

1. Can you please tell me about productivity measures that are important to the business in the growing herd?

*Probe:* Key performance indicators for the growing herd: Growth rate, mortality percentage, days to slaughter, feed efficiency.

a. From what you’ve discussed, which of the specific productivity measures are key determinants of profit?

*Probes: as above.*

2. How do meeting these productivity targets fit into the overall sustainability plan of the business/barn?

*Probe: The sustainability plan refers to the ability of the barn to remain profitable, maximize the performance potential of the animals, meet regulatory requirements for animal welfare, environmental targets, etc. [Or even have a plan to exceed national requirements for animal welfare and environmental targets, therefore taking strong leadership in a strategic business direction for sustainability].*

3. What do you consider are the main challenges to meeting productivity targets in the growing herd?

4. Can you please tell me about whether and if so, how, the indicators of animal welfare you’ve previously discussed relate to productivity and sustainability indicators? *If so, how, and why?*

5. Are there particular animal welfare indicators that directly relate to specific productivity and sustainability metrics?

*Probes: If needed, loop back to what the interviewee has spoken about with regards to practices and their influence on animal welfare, i.e., processing, space allowance, health management.*

6. Do you see any competing interests between meeting the animal welfare targets and production targets? Why or why not and can you elaborate?

# Great, thank you for the information. So, just a few final questions here.

7. We talked a lot about animal welfare in this interview. Overall, what do you see as the top three animal welfare challenges of the swine industry, or your barn, from your perspective?

a. Do you have any recommendations as to how to address these issues as an industry?

8. Overall, what do you see as the biggest worker welfare challenge of the swine industry from your perspective?

a. Do you have any recommendations as to how to address this issue as an industry?

9. Are there any additional comments you would like to add on anything we have discussed? Or anything else you think we should know?

**Thank you for taking the time to speak with us on this topic. We understand how valuable your time is and we are happy to have your input and experiences included in this study. We would like to come back and share our thoughts with you on what we have found from these interviews. Would you be happy to take a second final one hour call with us?**
